# Editor's Choice – Calcification of Thoracic and Abdominal Aneurysms is Associated with Mortality and Morbidity

**DOI:** 10.1016/j.ejvs.2017.11.007

**Published:** 2018-01

**Authors:** Mohammed M. Chowdhury, Lukasz P. Zieliński, James J. Sun, Simon Lambracos, Jonathan R. Boyle, Seamus C. Harrison, James H.F. Rudd, Patrick A. Coughlin

**Affiliations:** a Division of Vascular and Endovascular Surgery, Addenbrooke's Hospital, Cambridge University Hospital Trust, Cambridge, UK; b Division of Cardiovascular Medicine, University of Cambridge, Addenbrooke's Hospital, Cambridge, UK

**Keywords:** Atherosclerosis, Calcification, Thoracic abdominal aortic aneurysm, Computed tomography

## Abstract

**Introduction:**

Cardiovascular events are common in people with aortic aneurysms. Arterial calcification is a recognised predictor of cardiovascular outcomes in coronary artery disease. Whether calcification within abdominal and thoracic aneurysm walls is correlated with poor cardiovascular outcomes is not known.

**Patients and methods:**

Calcium scores were derived from computed tomography (CT) scans of consecutive patients with either infrarenal (AAA) or descending thoracic aneurysms (TAA) using the modified Agatston score. The primary outcome was subsequent all cause mortality during follow-up. Secondary outcomes were cardiovascular mortality and morbidity.

**Results:**

A total of 319 patients (123 TAA and 196 AAA; median age 77 [71–84] years, 72% male) were included with a median follow-up of 30 months. The primary outcome occurred in 120 (37.6%) patients. In the abdominal aortic aneurysm group, the calcium score was significantly related to both all cause mortality and cardiac mortality (odds ratios (OR) of 2.246 (95% CI 1.591–9.476; *p* < 0.001) and 1.321 (1.076–2.762; *p* = 0.003)) respectively. In the thoracic aneurysm group, calcium score was significantly related to all cause mortality (OR 6.444; 95% CI 2.574–6.137; *p* < 0.001), cardiac mortality (OR 3.456; 95% CI 1.765–4.654; *p* = 0.042) and cardiac morbidity (OR 2.128; 95% CI 1.973–4.342; *p* = 0.002).

**Conclusions:**

Aortic aneurysm calcification, in either the thoracic or the abdominal territory, is significantly associated with both higher overall and cardiovascular mortality. Calcium scoring, rapidly derived from routine CT scans, may help identify high risk patients for treatment to reduce risk.

**What this paper adds**

This is the first study to investigate the role of calcium scoring in aneurysmal aortic disease. A well validated assessment of arterial calcium scoring is described and demonstrates excellent reproducibility of score assessments within the arterial segments described. These data show high scores are associated with poor outcomes and lend weight to the possibility of calcium scoring in clinical practice as a predictive tool of poor cardiovascular outcomes in patients with aneurysmal disease.

## Introduction

Arterial calcification is now recognised as a significant marker of poor cardiovascular outcome. This is most evident within the coronary circulation. Coronary artery calcification can be quantified using the Agatston score; an independent predictor of future coronary events.^1^ Furthermore, the process atherosclerosis is a systemic inflammatory condition not only exclusive to the coronary or aortic circulation.

Arterial calcification is a systemic pathological process and as such can affect other arterial beds. Traditionally, calcification of both the abdominal and thoracic aorta has not been studied in the same detail as the coronary arterial circulation. Non-aneurysmal abdominal aortic calcification is strongly associated with both mortality and cardiovascular events rates with similar associations seen when considering the calcific burden of the thoracic aorta.^2^, ^3^

Both the abdominal and descending thoracic aorta are prone to aneurysmal degeneration. Abdominal aortic aneurysms are the most common form of aortic aneurysm, affecting 5% of the males aged 65–74.^4^ Thoracic aortic aneurysms have a more heterogeneous aetiology including atherosclerosis alongside less common genetic influences.

The emergence of endovascular intervention has revolutionised the management of aneurysmal disease, reducing the short-term risks associated with intervention.^5^ Such conditions however are still associated with significant long-term risk, specifically the risk of major cardiovascular events.^6^ As such, there is a continuing need for robust methods to predict such longer-term outcomes.

The evidence base for the predictive value of arterial calcification in aortic disease is limited, with only a small number of studies and no assessment in aneurysmal disease.^2^, ^7^ As such, and given the increasing use of CT based imaging modalities in the assessment of aneurysmal aortic disease, more in depth analysis of potential associations is both timely and warranted.

The primary aim of this study was to determine whether aortic aneurysmal calcification (the AAC score) could predict (a) all cause mortality and (b) cardiovascular related outcomes in a large, consecutive, well characterised cohort of patients with either thoracic and abdominal aortic aneurysms.

## Methods

### Patient identification

This was a retrospective, observational, single centre study consisting of consecutive asymptomatic patients undergoing CT angiography of either a thoracic aortic aneurysm (January 2008 to January 2012) or abdominal aortic aneurysm (January 2010 to January 2013), presenting to Addenbrooke's Hospital, Cambridge, UK. This study was registered with the local audit department to ensure all data collection was in line with local ethical standards. Radiology digital records were searched and all patients with a CT angiogram of the aorta identified. All CT scans were screened and an aneurysm diagnosis made when the largest single segment of the appropriate part of the aorta (infrarenal abdominal or thoracic) had an antero-posterior diameter of 3 cm or greater. Electronic patient records were reviewed to collect follow-up data, up to the final date of May 2015. Mortality and morbidity data were obtained from hospital medical records (cross-referenced with ONS data), hospital death certificates, and where further clarification was required general practitioners were contacted to access community medical notes, and community death certificates to ascertain the cause of death. Patients with a proven ruptured aneurysm or those imaged because of symptomatic aneurysms as well as patients with ascending or arch aneurysms were excluded (given the management of these patients in separate specialist cardiothoracic centre). Patients with aneurysms due to other causes (i.e., vasculitis) were also excluded. Patients with synchronous thoracic and abdominal aortic aneurysms were excluded to allow comparative analysis of calcification in these patients and to avoid confounding factors.

Patient demographics, medical history, and medication at the time of CT imaging were determined from the hospital electronic record system. Ischaemic heart disease (IHD) was defined as a clinical diagnosis of angina, a prescription of anti-anginal drugs, or a history of myocardial infarction or a coronary revascularisation procedure.

The primary endpoint was all cause mortality within the follow-up period with secondary endpoints of cardiac mortality and cardiac morbidity. Cardiac mortality was defined as death with documented evidence of acute myocardial infarction (MI). Cardiac morbidity was defined as a hospital admission with either (1) typical cardiac chest pain with ischaemic electrocardiogram (ECG) changes, or (2) typical chest pain with elevated cardiac enzymes, or (3) a discharge coding of a coronary event. Events were classified by the attending medical teams (in hospital event) or pathologists at post mortem (community event). Where hospital data were missing, data were sourced either from the primary care team or from the death certificate.

### Measurement of arterial calcification using the AAC score

Patients underwent CT imaging on a 64 slice CT scanner (Somatom Definition AS, Siemens, Crawley, UK), using standard clinical protocols. Briefly, scans were performed using helical acquisition with kV = 120, mA = 200, and a field of view of 350–380 mm yielding typical spatial resolution 0.7 × 0.7 × 3.0 mm^3^. Images were read and reported by consultant interventional vascular radiologists and maximum diameter was calculated using both AP diameters as well as double oblique reformats to the centreline. Diagnosis was based on enhanced CT imaging. ECG gating was not used in the thorax scans. From acquired raw data, the scan was reconstructed in 3 mm slices. Image analysis was performed on an Apple Macintosh computer (Apple Inc, Cupertino, CA, USA) using the open source DICOM viewer (v4, OsiriX Imaging Software, Pixmeo SARL).

**Figure 1:**
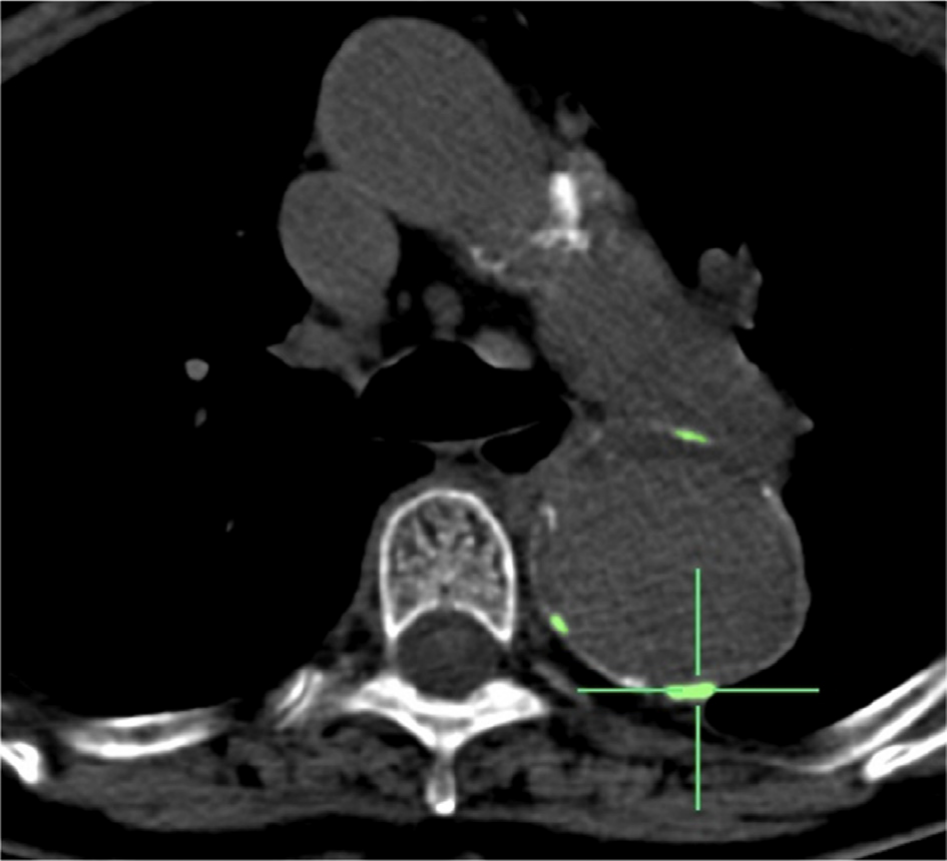
Calculation of calcium score using OsiriX. Example of thoracic aortic aneurysm scoring with scores derived from distal to left subclavian to upper aspect of coeliac axis using OsiriX.

Calcification data were derived from unenhanced CT images. Patients without unenhanced CT images were excluded. Using the freely available “Calcium Scoring” plug-in, vascular calcification (based on an attenuation threshold of 130 Hounsfield Units in 3 contiguous voxels, after the method of Agatston^8^) was analysed on consecutive trans-axial slices along the length of the arterial segment, as previously described^9^ (Fig. 1).

For the purposes of the thoracic aorta, the strict anatomical boundaries were from just distal to the left subclavian artery (proximal) to the proximal aspect of the coeliac axis (distal). For the abdominal aorta boundaries, analysis from the lowermost renal artery (proximal) down to the common iliac artery bifurcation on both limbs (distal) was performed. The AAC score was a cumulative score of each segment for both the thoracic aorta and the infrarenal abdominal aorta (including both aneurysmal and non-aneurysmal segments). No assessment of coronary calcium was made (inadequate and un-gated scans).

### AAC scoring reproducibility

Inter-observer reproducibility of the AAC scores was determined in 10 patients by two experienced readers (M.M.C. and L.P.Z./J.J.S., for both thoracic (J.J.S.) and abdominal (L.P.Z.) scans, respectively). Intra-observer reproducibility was determined for the AAC scores by an expert reader blinded to patient demographics (M.M.C.) for both thoracic and abdominal aneurysms. Fifteen unenhanced CT scans were scored on two occasions 7 days apart.

### Statistical methods

Statistical analyses were performed using SPSS (version 23, IBM, Armonk, NY, USA). The normality of continuous variables was checked using the Kolmogorov–Smirnov test; this demonstrated that AAC scores were non-parametric, which were logarithmically transformed for further analysis.^10^ Variables are expressed as mean ± standard deviation, median (Quartile 1 to Quartile 3) or *n* (%), as appropriate. Multivariate logistic regression adjusted analysis was performed to determine the individual parameters correlated with AAC. Receiver-operator characteristic (ROC) curves were generated for both scores against the primary outcome. Cutoff values were generated for equally weighted sensitivities and specificities. Intra- and inter-observer reproducibility was tested using intraclass correlation coefficients (ICC). A *p* value < 0.05 was considered statistically significant.

## Results

### Abdominal aortic aneurysm

A total of 196 patients with AAA were included in the study. Demographic data are shown in Table 1. The median age was 78 (72–84) years; 160 (81.6%) were male. Fifty-eight (30.0%) were current smokers, 46 (23.4%) had a diagnosis of IHD, and 21 (10.7%) prior cerebrovascular disease. The average maximum aneurysm diameter was 55 (47–66.3) mm. 103 (53%) patients underwent repair of their AAA during follow-up (85 endovascular, 18 open repair). The median AAAC score was 14,216 (7986–23,261). There was no significant difference between the operated and non-operated group (score, 14,911 vs. 13,978; *p* > 0.05, respectively).

**Table 1:** Baseline patient demographics (AAA).

	(*n* = 196)
Age, years	77 (69–83)
Male	160 (82)
Medical history at time of scan	
Ischaemic heart disease	46 (23)
Hypertension	59 (30)
Diabetes mellitus	18 (9)
Hypercholesterolaemia	43 (22)
Current smoker	58 (30)
Medications	
Antiplatelet agent	65 (33)
Statin use	32 (16)

Values are median (quartile 1 to quartile 3) or *n* (%).

There were 38 (19.4%) deaths during the follow up period of 22 (3–48) months. These included 10 patients who died because of myocardial infarction, two patients from left ventricular failure following an MI, and two gastrointestinal haemorrhages leading to fatal MI. Furthermore, there were 11 deaths attributed to pneumonia, seven end stage malignancy, two ruptured AAAs, and four with cause unknown. Only two deaths (MI and pneumonia) occurred within 30 days of operative intervention. Assessment of parameters, after correction for confounders (including age and sex) was by a multivariate regression analysis. The AAAC score was significantly related to the primary outcome with an OR 2.246 (95% CI 1.591–9.476; *p* < 0.001) (Table 2).

**Table 2:** Primary outcome: All cause mortality (AAAC group).

	Univariate logistic regression				Multivariate logistic regression			
	OR	95% CI		*p*	OR	95% CI		*p*
Age	**1.112**	**1.067**	**1.158**	<**0.001**	**1.084**	**1.032**	**1.138**	**0.001**
Male sex	1.857	0.895	3.852	0.096				
Diabetes	0.976	0.456	2.089	0.949				
HTN	1.689	0.884	3.228	0.113				
Smoker	0.835	0.453	1.540	0.564				
IHD/MI	0.881	0.482	1.612	0.681				
Antiplatelet	**2.134**	**1.027**	**4.433**	**0.042**	0.625	0.202	1.934	0.415
Statin	**2.346**	**1.224**	**4.496**	**0.010**	1.427	0.526	3.873	0.486
Diameter	0.978	0.810	1.181	0.817				
Ca^2+^ score	**3.706**	**2.641**	**9.591**	<**0.001**	**2.246**	**1.591**	**9.476**	<**0.001**

*Note*. Significance is highlighted in bold. HTN = hypertension; IHD/MI = ischaemic heart disease/myocardial infarction; OR = odds ratio; CI = confidence interval.

Cardiac mortality occurred in 14 patients (7.1%) and cardiac morbidity in 20 patients (10.2%; comprising of 17 patients diagnosed with a MI, 2 patients with decompensated heart failure and 1 patient admitted with unstable angina). AAAC score was predictive of cardiac mortality (OR 1.321 [1.076–2.762]; *p* = 0.003) (Table 3), but despite a clear correlation of pre-existing cardiac disease and cardiac morbidity (*p* = 0.001), there was no correlation between calcium score and cardiac morbidity (OR 1.231; 95% CI 0.872–1.453; *p* = 0.341).

**Table 3:** Secondary outcome: Cardiac mortality (AAAC group).

	Univariate logistic regression				Multivariate logistic regression			
	OR	95% CI		*p*	OR	95% CI		*p*
Age	1.065	1.021	1.137	0.048	1.232	0.987	1.982	0.078
Male Sex	0.725	0.155	3.393	0.683			–	–
Diabetes	0.753	0.198	2.857	0.676			–	–
HTN	1.643	0.524	5.152	0.395			–	–
Smoker	0.624	0.207	1.880	0.402			–	–
IHD/MI	0.689	0.229	2.074	0.507			–	–
Antiplatelet	1.231	0.325	4.661	0.759			–	–
Statin	2.283	0.752	6.934	0.145			–	–
Diameter	1.237	0.897	1.707	0.195			–	–
Ca^2+^ score	**1.192**	**1.075**	**2.472**	**0.002**	**1.321**	**1.076**	**2.762**	**0.003**

*Note*. Significance is highlighted in bold. HTN = hypertension; IHD/MI = ischaemic heart disease/myocardial infarction; OR = odds ratio; CI = confidence interval.

No documented complications were encountered due to common femoral/access points or further infrainguinal arterial calcification.

**Figure 2:**
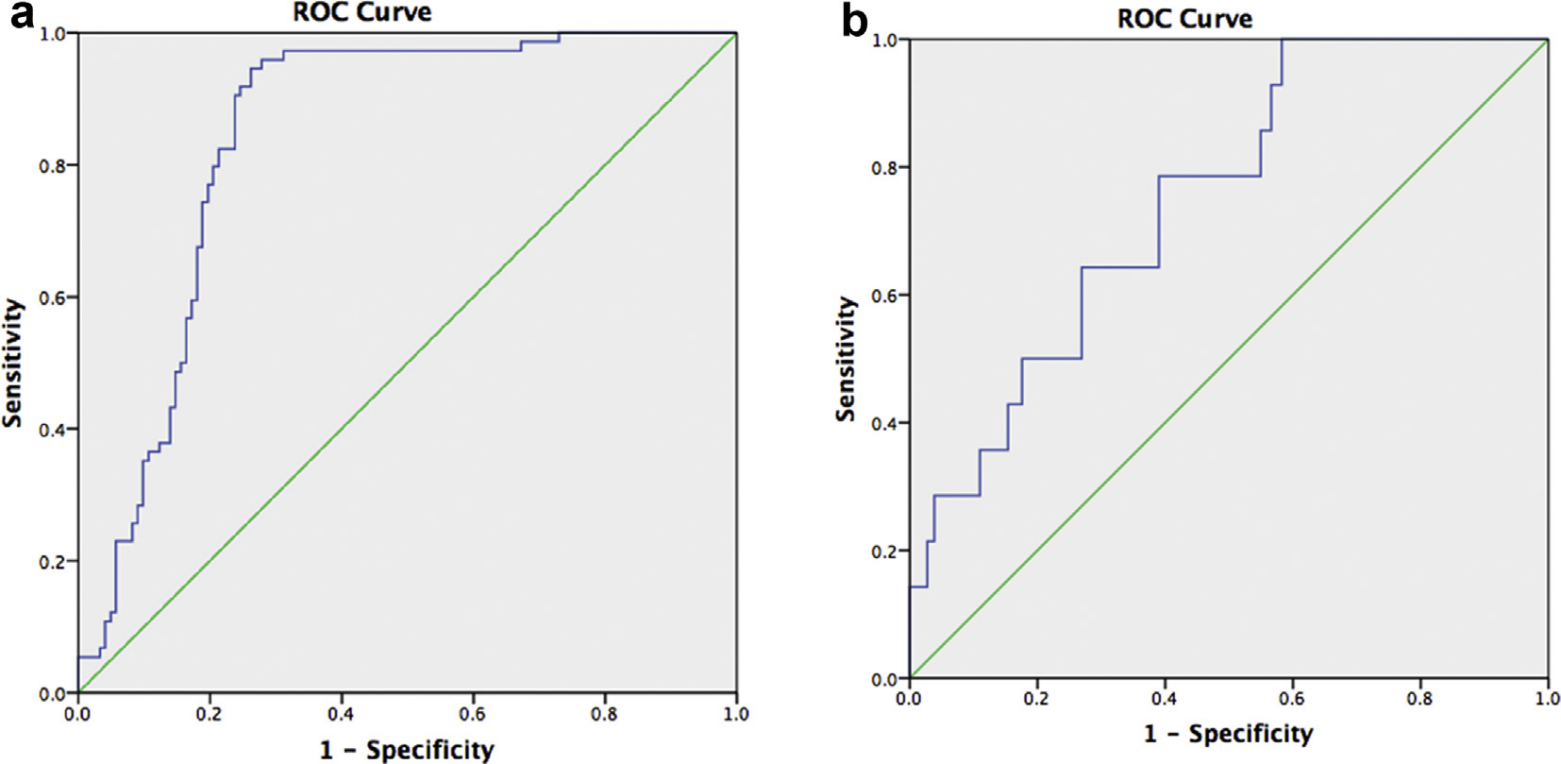
(a) ROC analysis – all cause mortality (abdominal aortic aneurysms). AUC for the AAC score was 0.815 (95% CI 0.755–0.875; *p* < 0.001). (b) ROC analysis – cardiac mortality (abdominal aortic aneurysms). AUC for the score was 0.748 (95% CI 0.633–0.864; *p* = 0.02). AAC = aneurysmal aortic calcium; ROC = receiver-operator curve; AUC = area under the curve; CI = confidence interval.

ROC analysis curves were generated to test the power of the AAAC score to predict all cause mortality and the secondary outcomes (Fig. 2a and b). For all cause mortality, an AUC value of 0.815 (95% CI 0.755–0.875; *p* < 0.001) demonstrated that a cutoff value of >21,000 (at a sensitivity of 84%, specificity of 74.6%) would predict the outcome. For cardiac mortality, an AUC value of 0.748 (95% CI 0.633–0.864; *p* = 0.02) demonstrated that a cutoff value of >22,500 (at a sensitivity of 78.6%, specificity of 68.3%) would predict the outcome.

Inter- and intra-observer reliability scores for AAC were ICC 0.93 and 0.95, respectively.

### Thoracic aortic aneurysm

A total of 123 patients with thoracic aneurysms were included in the study. The median age of this cohort was 77 (70–83) years; 74 (60%) were male. Demographic data are shown in Table 4. Forty-one (33%) were current smokers, 64 (52%) had pre-existing IHD and 23 (19%) prior cerebrovascular disease. The average maximum aneurysm diameter was 62 (53–67.5) mm, with 75 (61%) patients undergoing endovascular repair during the follow up period. No patients underwent open repair. The median TAAC score for all patients was 6263 (1168–16,165). There was no significant difference between the operated and non-operated group (score 5296 vs. 6210; *p* > 0.05, respectively).

**Table 4:** Baseline patient demographics (thoracic aortic aneurysms).

	(*n* = 123)
Age, years	78 (72–84)
Male	74 (60)
Medical history at time of scan	
Ischaemic Heart Disease	42 (34)
Hypertension	41 (33)
Diabetes Mellitus	21 (17)
Hypercholesterolaemia	51 (41)
Current smoker	41 (33)
Medications	
Antiplatelet agent	53 (43)
Statin use	48 (39)

Values are median (quartile 1 to quartile 3) or *n* (%).

During follow-up of 45 (10–55) months, there were 46 (37%) deaths. The leading causes were myocardial infarction (*n* = 10), acute left ventricular failure (*n* = 2), gastrointestinal haemorrhage leading to a myocardial infarction (*n* = 1), ruptured aneurysm (*n* = 9), pneumonias (*n* = 7), malignancy (*n* = 6), intracerebral bleed (*n* = 1), and vascular dementia in one patient. In nine patients, the cause of death was not determined. Only two deaths (MI) occurred within 30 days of operative intervention. Again, following multivariate regression analysis, the TAAC score was significantly related to the primary outcome (OR 6.444; 95% CI 2.574–6.137; *p* < 0.001) (Table 5).

**Table 5:** Primary outcome: All cause mortality (TAAC group).

	Univariate logistic regression				Multivariate logistic regression			
	OR	95% CI		*p*	OR	95% CI		*p*
Age	**1.054**	**1.014**	**1.095**	**0.007**	1.016	0.974	1.061	0.460
Male Sex	0.907	0.431	1.910	0.797				
Diabetes	1.613	0.578	4.503	0.362				
HTN	1.046	0.485	2.257	0.908				
Smoker	0.901	0.417	1.950	0.792				
IHD/MI	0.704	0.328	1.512	0.368				
Antiplatelet	0.738	0.354	1.535	0.416				
Statin	1.265	0.608	2.631	0.530				
Diameter	0.748	0.551	1.017	0.064				
Ca^2+^ Score	**6.054**	**2.845**	**6.898**	<**0.001**	**6.444**	**2.574**	**6.137**	<**0.001**

*Note*. Significance is highlighted in bold. HTN = hypertension; IHD/MI = ischaemic heart disease/myocardial infarction; OR = odds ratio; CI = confidence interval.

Cardiac mortality occurred in 13 patients (11%) and cardiac morbidity in 17 patients (14%; comprising 15 patients with a diagnosis of MI and 2 patients with a diagnosis of unstable angina). TAAC score was predictive of cardiac mortality (OR 3.456 [1.765–4.654]; *p* = 0.042) (Table 6), and cardiac morbidity (OR 2.128; 95% CI 1.973–4.342; *p* = 0.002) (Table 7).

**Table 6:** Secondary outcome: Cardiac mortality (TAAC group).

	Univariate logistic regression				Multivariate logistic regression			
	OR	95% CI		*p*	OR	95% CI		*p*
Age	1.021	0.968	1.077	0.444				
Male sex	1.558	0.452	5.370	0.483				
Diabetes	2.667	0.328	21.706	0.359				
HTN	1.546	1.142	2.103	0.034	1.765	0.987	2.543	0.087
Smoker	0.383	0.120	1.227	0.106				
IHD/MI	0.811	0.248	2.653	0.729				
Antiplatelet	0.581	0.179	1.888	0.367				
Statin	0.478	0.139	1.645	0.242				
Diameter	0.844	0.530	1.346	0.477				
Ca^2+^ score	**4.790**	**1.102**	**20.817**	**0.037**	**3.456**	**1.765**	**4.654**	**0.042**

*Note*. Significance is highlighted in bold. HTN = hypertension; IHD/MI = ischaemic heart disease/myocardial infarction; OR = odds ratio; CI = confidence interval.

**Table 7:** Secondary outcome: Cardiac morbidity (TAAC group).

	Univariate logistic regression				Multivariate logistic regression			
	OR	95% CI		*p*	OR	95% CI		*p*
Age	1.047	0.991	1.106	0.105	–	–	–	–
Male Sex	0.407	0.144	1.157	0.092	–	–	–	–
Diabetes	0.955	0.248	3.668	0.946	–	–	–	–
HTN	0.551	0.168	1.808	0.325	–	–	–	–
Smoker	0.904	0.309	2.645	0.853	–	–	–	–
IHD/MI	1.531	1.189	2.497	0.023	1.672	1.098	1.897	0.345
Antiplatelet	0.649	0.230	1.833	0.415	–	–	–	–
Statin	0.433	0.143	1.314	0.139	–	–	–	–
Diameter	1.245	0.822	1.886	0.300	–	–	–	–
Ca^2+^ score	**9.609**	**4.079**	**22.639**	**0.005**	**2.128**	**1.973**	**4.342**	**0.002**

Note. Significance is highlighted in bold. HTN = hypertension; IHD/MI = ischaemic heart disease/myocardial infarction; OR = odds ratio; CI = confidence interval.

No documented complications were encountered due to common femoral/access points or further infrainguinal arterial calcification.

**Figure 3:**
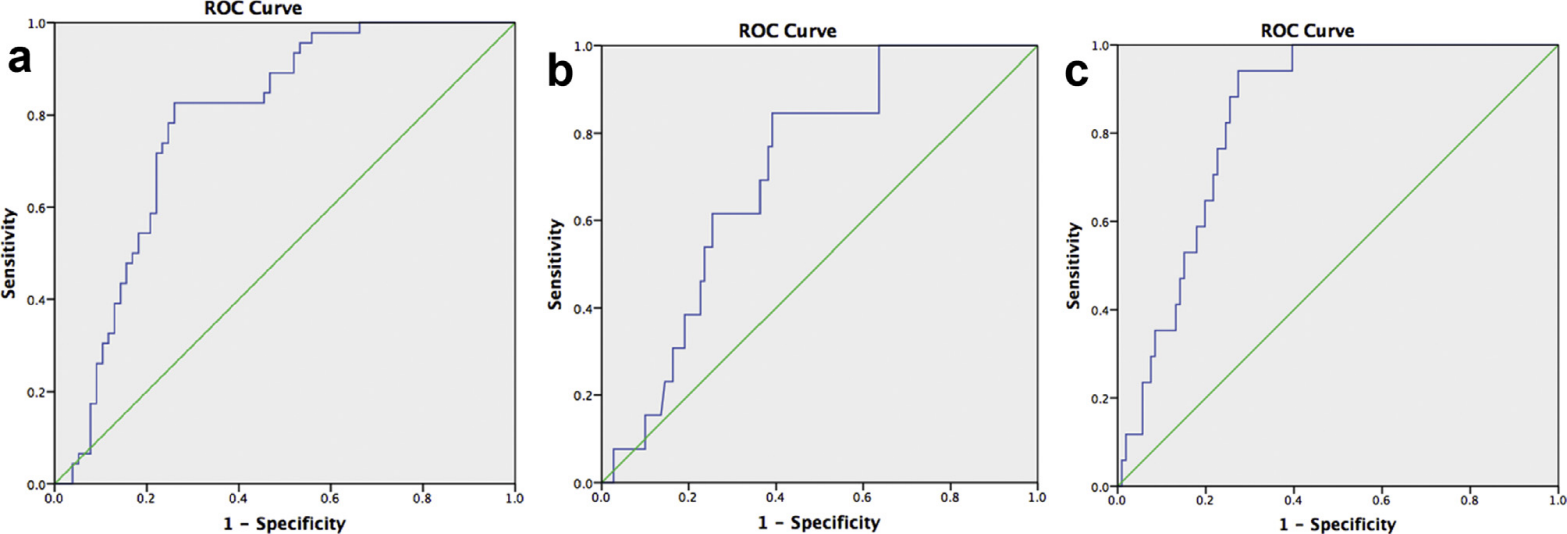
(a) ROC analysis – all cause mortality (thoracic aortic aneurysm). AUC for the AAC score was 0.785 (95% CI 0.704–0.865; *p* < 0.001). (b) ROC analysis – cardiac mortality (thoracic aortic aneurysm). AUC for the AAC score was 0.712 (95% CI 0.595–0.828; *p* = 0.013. (c) ROC analysis – cardiac morbidity (thoracic aortic aneurysm). AUC for the AAC score was 0.840 (95% CI 0.768–0.912; *p* < 0.001). AAC = aneurysmal aortic calcium; ROC = receiver-operator curve; AUC = area under the curve; CI = confidence interval.

ROC analysis curves were generated to test the power of the AAC score to predict all cause mortality and both secondary outcomes (Fig. 3a–c). For all cause mortality, an area under the curve (AUC) value of 0.785 (95% CI 0.704–0.865; *p* < 0.001) demonstrated that a cutoff value of >5800 (at a sensitivity of 82.6%, specificity of 73.4%) would predict the outcome. For the secondary outcomes, cardiac mortality (AUC 0.712; 95% CI 0.595–0.828; *p* = 0.013) a cutoff value of >5500 (at a sensitivity of 84.6%, specificity of 75.6%) would predict the outcome, whereas with cardiac morbidity (AUC 0.840; 95% CI 0.768–0.912; *p* < 0.001)) a cutoff value of >6100 (at a sensitivity of 82.4%, specificity of 79.8%) would predict the outcome.

## Discussion

In this study, calcification of both the aneurysmal thoracic and the infrarenal abdominal aorta was predictive of all cause mortality. Furthermore, these relationships remained significant after correcting for potential confounders (such as age and blood pressure) in a multivariate regression analysis. In addition, AAC was correlated with adverse cardiac specific mortality irrespective of site of aneurysmal disease. Quantitative assessment of AAC score, using computed tomography, was feasible, highly reproducible, and has been proven to be a useful surrogate for long-term clinical outcomes. Interestingly, after regression analysis assessing the predictive power of aneurysm size and calcium score, it was only score that proved significant to predict the outcomes, irrespective of aneurysm size. Specifically, this study also provides appropriate cutoff values for calcium scoring that predict patient relevant outcomes for both the thoracic and abdominal aorta. These now require further prospective validation.

The endovascular revolution has transformed how aneurysm disease is now managed. Increasingly, endografting is the first line treatment.^11^ Within the abdominal aorta this move towards endovascular aortic repair (EVAR) is associated with an early survival advantage that is subsequently eroded at 5 years.^12^ However, such short-term benefits have encouraged the treatment of older patients with greater comorbidity.^13^ Pre-operative assessment and risk stratification is key in these patients; the ability to be build a robust operative risk tool, perhaps with the inclusion of calcium score, could help identify patients at higher risk of post-operative complications.

Aneurysm calcium scoring could have a role in risk stratification of patients (in particular when assessing patient fitness and disease burden pre-operatively). The process of scoring is a quick and reproducible technique that could easily be employed in clinical practice (similar to coronary scoring). OsiriX is freely available software and there is an easy to use plug-in which allows the scoring of calcium from CT images. As reported, intra- and inter-observer variability was minimal. The approximate time to score the aneurysm was 3 min per scan.

The data are less strong when assessing thoracic aortic aneurysms. No randomised controlled trials have compared thoracic EVAR (TEVAR) with conventional open surgical repair, yet a recent Cochrane review concluded that TEVAR was associated with a reduction in early outcomes including paraplegia, mortality, and hospital stay. A lack of available high quality data limited the conclusions with regard to longer-term outcomes.^14^ It is recognised that patients with aneurysm disease have significant cardiovascular risk and that there is an association with increasing diameter.^15^ Here, it is shown that calcium score is also an independent risk factor of overall cardiovascular risk.

Given the increasing use such interventions, specifically within the older patients, as well as the overall associated cardiovascular event risk, there is a continuing need to identify those patients with an overall reduced life expectancy. This will allow focused cardiovascular risk factor management in such patients as well as enhancing the decision making process when an aneurysm reaches the size threshold for intervention. This study shows that the burden of aortic calcification is an accurate predictor of poor patient outcome. It is a clinically relevant measure, as all patients being considered for intervention will undergo CT evaluation. The determination of calcification is straightforward to perform and excellent reliability has been shown.

Calcification occurs in response to chronic inflammation, a key process in the development of aortic aneurysm disease. Calcification seen in the aorta is found within the intimal and medial layers of the arterial wall. In contrast, there is predominantly intimal calcification seen within the atherosclerotic plaques in the coronary arteries. Medial calcification is strongly associated with aging, diabetes and end stage disease.^16^ Yet evidence suggests that calcification occurs differently within differing arterial beds, with the associations between calcified atherosclerosis and mortality also different depending on vascular bed.^17^ It is thought that the calcium deposition in the vessel wall leads to vascular stiffness and subsequent hypertension.^18^ The association between coronary artery calcification and mortality is clear.^19^ In the non-aneurysmal aorta, thoracic aortic calcification is associated with total mortality and all cardiovascular events whereas abdominal aortic calcification is associated more with cardiovascular mortality and morbidity. When other arterial beds are considered, there has been shown to be marked variability in degree of calcification and patient outcomes suggesting that the location and severity of calcification provides unique information for mortality.^10^

In this study, mortality due to cardiovascular causes occurred in up to 10% of the study cohort. Pneumonia was also a cause of death in a substantial number of cases and may well reflect patients with poor respiratory reserve in keeping with a significant smoking history, a further risk for arterial calcification. Interestingly, there was no significant correlation between calcium score and cardiac morbidity. This may, in part, be due to low outcome numbers and generally small sample sizes. This lends further weight to the role of calcium scoring as a predictor of poor outcomes given that such patients may not have been identified as previously needing aggressive secondary cardiovascular risk prevention. Why do patients with increased arterial calcification have higher rates of morbidity and mortality? In part, this may reflect a generalised lower grade chronic inflammatory process which has been associated with a number of diseases associated with ageing.^20^, ^21^ It may also be that patients with AAAs have associated atherosclerotic risk factors and therefore have an increased prevalence of coronary heart disease and risk of cardiovascular events.^22^, ^23^ This probably reflects the demographics of AAA patients who tend to be older men with a history of smoking. Arterial calcification itself, specifically within the aorta, is likely to contribute to arterial stiffening and subsequent hypertension.^24^ This undoubtedly is a potential aetiological factor in the development of cardiovascular morbidity and mortality.

### Limitations

This study was a retrospective study, although a complete dataset was obtained. A pragmatic study design was used and as such included all patients with an asymptomatic aneurysm irrespective of whether they underwent surgical intervention or not, so some patients did succumb to a ruptured aneurysm within the follow up, although these numbers were small. Aneurysms of the ascending thoracic aorta and arch as well as symptomatic aneurysms were not included in the analysis. This was a CT specific study to allow for calcium scoring and so the study cohort reflects (a) those patients with an AAA reaching threshold for treatment and (b) patients undergoing treatment/surveillance with a thoracic aneurysm. It therefore doesn't reflect patients undergoing ultrasound surveillance for a small AAA or those patients being assessed using magnetic resonance angiography (MRA), although MRA is little used within the practice. As such, those patients with the most severe comorbidity in whom a decision not to treat was made without CT imaging may well not be represented within this cohort of patients. Specifically renal dysfunction was not analysed, a recognised cause of calcification, given that the lack of CT angiography in such patients due to the perceived risk of contrast nephropathy may well have biased the results. As such, this study is reflective of clinical practice. It would also be useful to investigate the link between aneurysmal calcium score and coronary artery calcium score. Furthermore, the assessment of calcium burden was not correlated with the presence of peripheral arterial disease (using ankle brachial pressure index; ABPI). There may be merit in assessing this correlation with further studies, given its association with cardiovascular morbidity and mortality. ABPI data were not available for this cohort of patients.

In current practice, use of non-contrast CT of the chest is rapidly emerging as a screening tool for identifying asymptomatic patients at high risk of heart disease, as well as having a role in lung cancer screening.^25^

## Conclusion

Given the routine use of CT angiography in the assessment of patients with aneurysmal disease, the role of calcium scoring in aneurysmal disease has potential to help identify patients at higher risk of longer-term morbidity and mortality, and this warrants prospective validation. The data identify a cohort of patients with increased risk of poor outcomes. Further understanding of the calcification process may help to improve cardiovascular outcomes.

## Conflict of interest

None.

## Funding

M.M.C. is supported by the Royal College of Surgeons of England Fellowship Programme and a British Heart Foundation Research Fellowship award FS/16/29/31957. P.A.C. is supported by British Heart Foundation, Circulation Foundation and Dunhill Medical Trust. J.H.F.R. is part supported by the NIHR Cambridge Biomedical Research Centre, the British Heart Foundation, the EPSRC and the Wellcome Trust.
